# Analyzing the Distribution and Antifungal Susceptibility Profile of Candida in Various Clinical Samples: A Retrospective Study From a Tertiary Care Hospital in North India

**DOI:** 10.7759/cureus.90131

**Published:** 2025-08-15

**Authors:** Amit Kumar, Nishtha Singh, Anupam Das, Jyotsana Agarwal

**Affiliations:** 1 Microbiology, Autonomous State Medical College, Kanpur Dehat, IND; 2 Microbiology, Hind Institute of Medical Sciences, Barabanki, IND; 3 Microbiology, Dr. Ram Manohar Lohia Institute of Medical Sciences, Lucknow, IND

**Keywords:** antifungal susceptibility, candida auris, candidemia, invasive fungal infection, nosocomial infection

## Abstract

Introduction

*Candida* is a leading cause of nosocomial infections due to its invasive nature and presence in prosthetic devices. *Candida albicans* is the most common *Candida* species, but prevalence varies by location. Globally, there has been a shift toward non-albicans *Candida* (NAC). The study aimed to determine the distribution of *Candida* species across various clinical samples.

Materials and methods

From January to December 2024, we conducted a retrospective observational study at Dr. Ram Manohar Lohia Institute of Medical Sciences (RMLIMS) Hospital in Lucknow. We reviewed antifungal resistance and patient medical information using the hospital information system. According to a database review, 1,641 *Candida* isolates were identified from a year's worth of clinical samples. For routine analysis, the clinical samples were processed following standard protocols. The Vitek 2 system (BioMérieux SA, Marcy-l'Étoile, France) was used to determine the antifungal susceptibility testing patterns of the isolates, while the matrix-assisted laser desorption/ionization time-of-flight (MALDI-TOF) mass spectrometry (MS) (BioMérieux SA, Marcy-l'Étoile, France) was employed for species-level identification.

Results

Urine made up 772 (47.1%) of all samples, followed by respiratory samples at 445 (27.1%) and blood at 387 (23.6%). Twelve distinct species of *Candida* were isolated. Compared to *Candida albicans* 450 (27.4%), NAC species accounted for 1191 (72.6%) of the total. *Candida tropicalis* was the most often isolated species of NAC. Among all *Candida *species, fluconazole was found to be the most resistant antifungal agent.

Conclusion

NAC species were responsible for a notable epidemiological shift in cases of candidiasis. Regularly identifying *Candida *isolates at the species level and, whenever possible, using antifungal susceptibility testing to identify emerging resistant strains is crucial for management.

## Introduction

Fungi are emerging with a more resistant pattern due to environmental and human behavior. The majority of fungal infections, which affect around 1.7 billion people globally, are superficial infections of the skin and mucosal membranes [1]. *Candida* species rank fourth in terms of all hospital-acquired infections and are considered to be the primary source of nosocomial fungal infections [2]. Worldwide, *Candida* species cause around 400,000 bloodstream infections annually, with death rates of over 40% [3]. Non-albicans species such as *Candida tropicalis*, *Candida parapsilosis*, and *Candida glabrata* have increased in frequency in recent decades. Resistance to the azole group is more commonly found in species other than *Candida albicans*, despite the fact that this is the most often seen species [4].

*Candida* species are the most common opportunistic infections in individuals who have compromised immune systems with decreased physiological and cellular barriers. Their ability to exist in two forms, yeast and filamentous forms, helps them to stick to host tissues and medical equipment, penetrate tissues, form biofilms, and release extracellular hydrolytic enzymes. Their characteristic feature of existing in two forms makes them more pathogenic [5,6]. The extensive use of corticosteroids, antifungal medications, and broad-spectrum antibiotics has enhanced their survival. Together with regional variations in epidemiology, this has led to a rise in their occurrence, distribution, and variance [7].

Due to its high mortality rate, antifungal resistance, and propensity for breakout, *Candida auris* infection has become a significant problem among the public. The treatment of candidemia infection in hospitalized patients has become a challenge. Before the discovery of "index" cases of clinical infection, *Candida auris* probably spread from patient to patient [8]. *Candida auris* typically goes undetected for days or weeks following hospitalization, which gives the fungus an opportunity to proliferate in medical facilities [9]. Formation of "dry" biofilms led *Candida auris* to survive on equipment and environmental surfaces for several months.

Characterizing *Candida* species at the initial level is crucial for identifying strains that may be intrinsically resistant to antifungal agents, such as *Candida krusei* to fluconazole [10]. The misuse of antifungal agents has led to increased resistance among *Candida albicans* and non-albicans species, thereby posing serious challenges for the physician to give treatment for *Candida* infections [11]. Variations and distribution in *Candida* species in various samples and their susceptibility to different antifungal agents are of utmost importance in early diagnosis and managing these infections. The study's objective was to retrospectively analyze data on the variety of *Candida* species, their distribution in different clinical samples, and their susceptibility profile at a tertiary care hospital in North India.

## Materials and methods

From January to December 2024, we conducted a retrospective observational study at the Dr. Ram Manohar Lohia Institute of Medical Sciences (RMLIMS) Hospital in Lucknow. The study's goal was to determine the distribution of *Candida* species across different clinical samples. The data are collected from our hospital information system (HIS). Patient demographics (gender, age), hospital wards, outpatient services, specimen type, outcomes of speciation, and antifungal susceptibility pattern were all included in the database. All clinical samples that showed the growth of *Candida* species were included in the study. Urine, blood, respiratory samples (sputum, bronchoalveolar lavages (BAL), endotracheal (ET) aspirate), body fluids, and pus were among the various samples collected. Duplicate samples from the same patient and cultures with mixed growths were excluded.

The clinical samples were processed according to usual procedures after being received for regular analysis. Aseptic methods were used to collect all blood culture samples in blood culture bottles, which were then incubated in BacT/Alert 3D (BioMérieux SA, Marcy-l'Étoile, France). When the bottle flagged positive, it was subcultured on blood agar (HiMedia Laboratories Pvt. Ltd., Mumbai, India) and MacConkey agar (HiMedia Laboratories Pvt. Ltd., Mumbai, India), and plates were incubated overnight at 37°C. Gram staining was done on the colony. Other samples were first inoculated in a Sabouraud dextrose agar (SDA) tube and incubated at 37°C. SDA tube readings were performed to monitor growth. Upon growth in the SDA tube, Gram staining is performed. After the identification of fungal organisms from Gram stain, the isolates were identified down to the species level using the matrix-assisted laser desorption/ionization time-of-flight (MALDI-TOF) mass spectrometry (BioMérieux SA, Marcy-l'Étoile, France), and their antifungal susceptibility testing (AFST) patterns were ascertained using the Vitek 2 system (BioMérieux SA, Marcy-l'Étoile, France). Amphotericin B, flucytosine, caspofungin, micafungin, fluconazole, and voriconazole were among the antifungals on the AFST panel. The Clinical Laboratory Standards Institute's (CLSI) 2023 standards were followed in classifying the susceptibility data as sensitive (S), intermediate (I), or resistant (R).

The data gathered for this investigation were analyzed using the statistical program SPSS Statistics version 26 (IBM Corp., Armonk, NY). Frequencies for categorical variables were computed for descriptive purposes. To examine the differences and determine the relationship between the variables, Pearson's chi-square test was employed. Depending on the distribution of the continuous variable, either means or medians were reported. When a p-value was less than 0.05, the difference was deemed statistically significant. The institutional ethics committee's approval was not necessary, as it is a retrospective study and does not involve patients directly.

## Results

**Figure 1:**
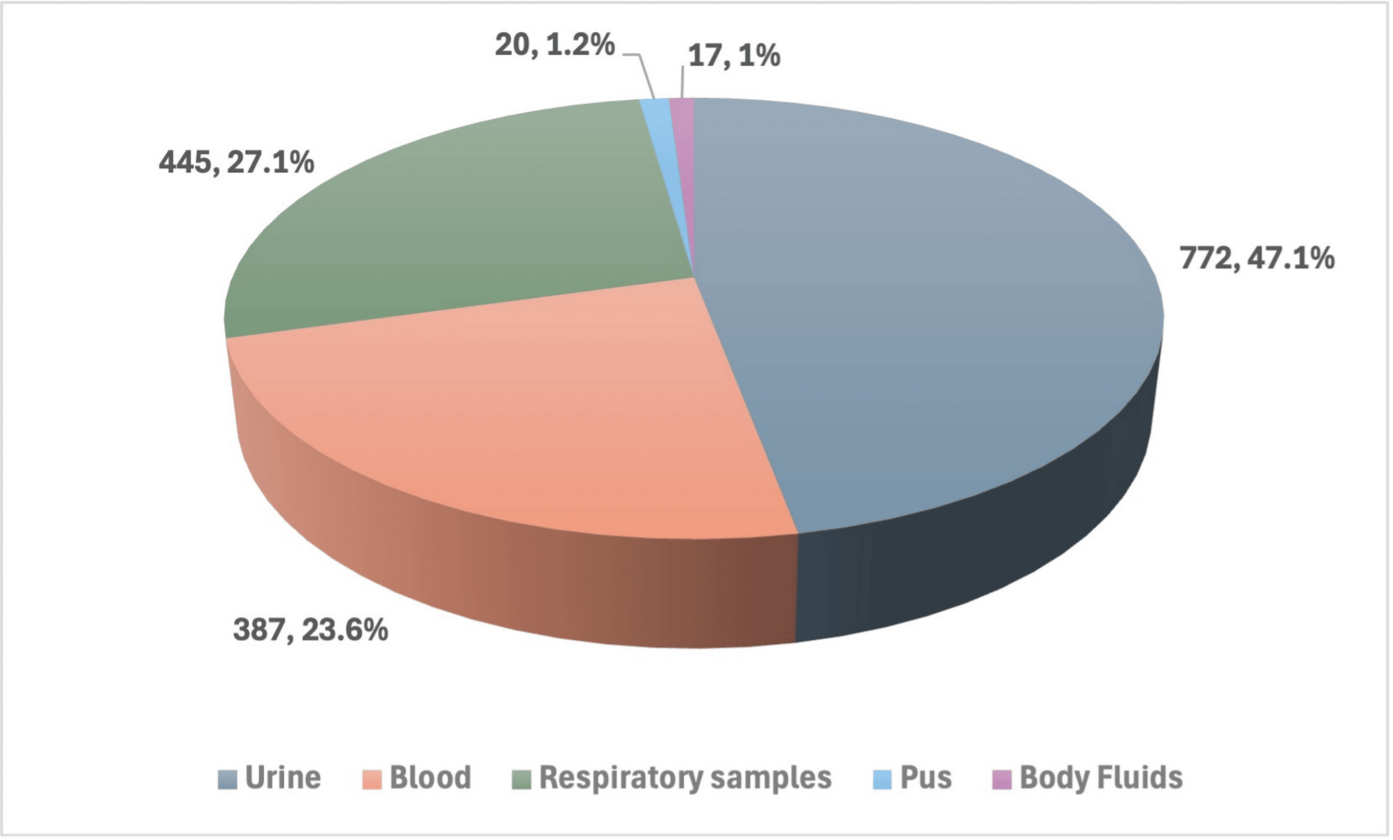
Distribution of different clinical samples

In our study, 1,641 *Candida* isolates were discovered from different clinical samples over one year. Sputum, BAL, ET aspirates, urine, blood, and other bodily fluids were among the processed clinical samples. Urine comprised (n = 772, 47.1%) of all samples, followed by respiratory specimens at (n = 445, 27.1%) and blood at (n = 387, 23.6%) (Figure 1).

**Figure 2:**
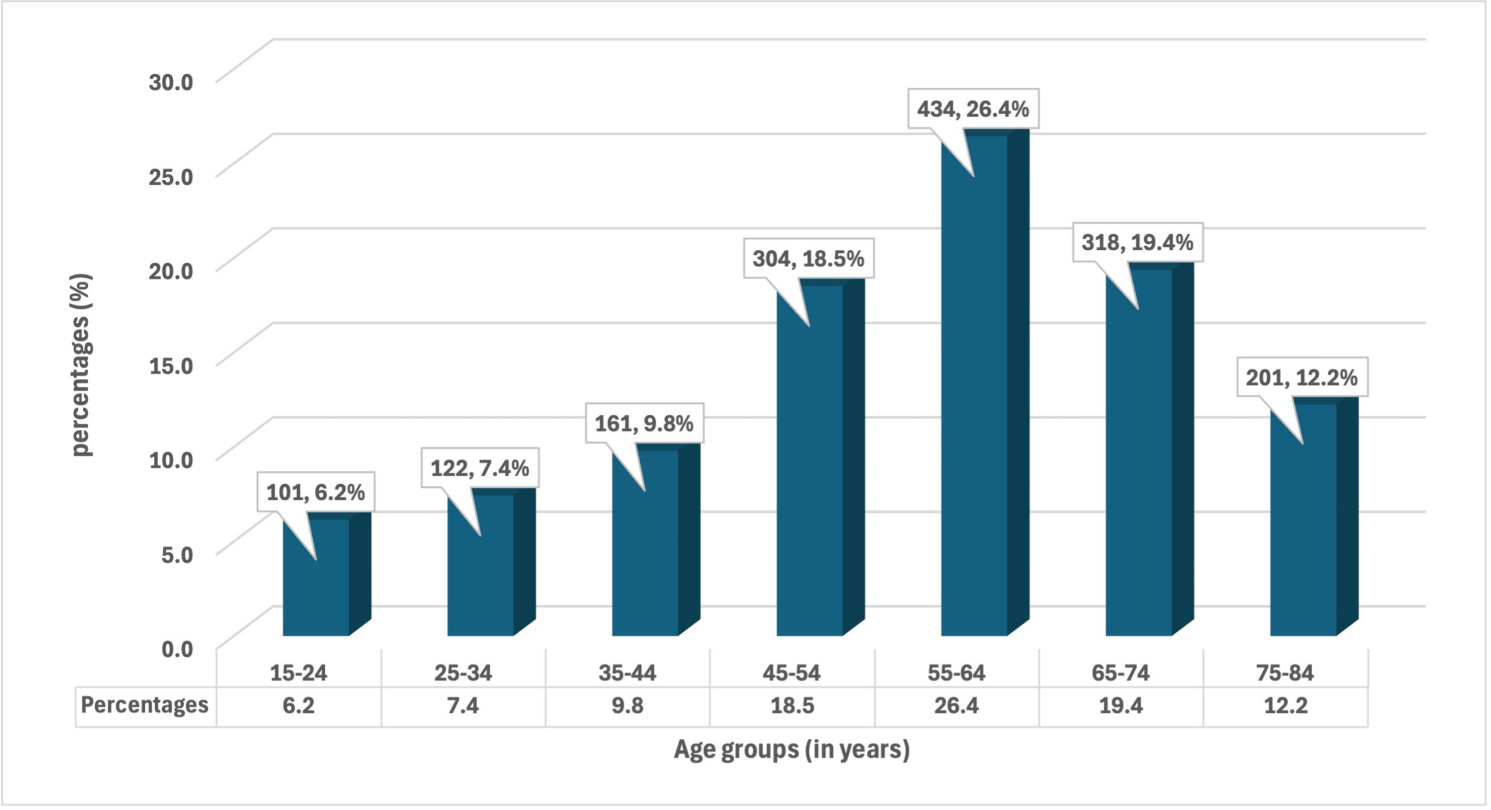
Distribution of patients according to age group

*Candida* infections were more common in females (n = 945, 57.6%) compared to males (n = 696, 42.4%), resulting in a ratio of 1.36:1. In our research, the most commonly affected age group was 55-64 years (n = 434, 26.4%), followed by 65-74 years (n = 318, 19.4%), with a mean age of 55.4 years (Figure 2).

Out of the 1,641 isolates, 12 distinct species of *Candida* were recovered, among which *Candida tropicalis* was the most frequently isolated species, accounting for 36.50% (n = 599; 95% CI: 34.17-38.83) of the cases. This was followed by *Candida albicans*, comprising 27.4% (n = 450; 95% CI: 25.26-29.58). Less frequently encountered species were *Candida rugosa* (0.37%; n = 6), *Candida kefyr* and *Candida utilis* (each 0.30%; n = 5), *Candida orthopsilosis* (0.24%; n = 4), and *Candida lusitaniae* (0.06%; n = 1). These findings highlight the predominance of non-albicans *Candida* (NAC) species, particularly *Candida tropicalis*, in fungal isolates (Table 1).

**Table 1:** Overall distribution of fungal isolates

Fungal isolates	Number (n)	Percentages (%)	95% CI
Candida albicans	450	27.42	25.26-29.58
Candida auris	74	4.51	3.51-5.51
Candida glabrata	104	6.34	5.16-7.52
Candida kefyr	5	0.30	0.04-0.57
Candida krusei	276	16.82	15.01-18.63
Candida lusitaniae	1	0.06	0.0-0.18
Candida orthopsilosis	4	0.24	0.01-0.48
Candida parapsilosis	72	4.39	3.40-5.38
Candida pelliculosa	45	2.74	1.95-3.53
Candida tropicalis	599	36.50	34.17-38.83
Candida utilis	5	0.30	0.04-0.57
Candida rugosa	6	0.37	0.07-0.66
Total	1641	100.00	-

Among the 1,641 fungal isolates analyzed, the distribution across hospital settings - ICU, inpatient department (IPD), and outpatient department (OPD) - showed statistically significant variation for several species. *Candida tropicalis* was the most common isolate overall (36.50%), with the highest numbers from the IPD (n = 284; 17.31%), followed by the ICU (n = 202; 12.31%), though this variation was not statistically significant (p = 0.262). In contrast, *Candida auris* demonstrated a highly significant difference (p < 0.001), being more prevalent in ICU (n = 56; 3.41%) compared to IPD (n = 14; 0.85%). Similarly, *Candida glabrata* showed significant variation (p < 0.001), with equal counts in ICU and IPD (n = 43 each; 2.62%) but fewer in OPD (n = 18; 1.10%). *Candida pelliculosa* also differed significantly across groups (p < 0.001), mostly found in IPD (n = 41; 2.50%). Some other isolates, like *Candida parapsilosis*, showed modest but statistically significant differences (p = 0.041), whereas variations in *Candida albicans*, *Candida krusei*, and others were not statistically significant (p > 0.05). These data indicate that while certain *Candida species* are distributed evenly across clinical settings, others, like *Candida auris*, *Candida glabrata*, and *Candida pelliculosa*, have distinct patterns, particularly with higher occurrence in ICU or IPD environments (Table 2).

**Table 2:** Distribution of fungal isolates according to the type of wards ICU = intensive care unit, IPD = inpatient department, n = number, OPD = outpatient department, % = percentages

Fungal isolates	ICU		IPD		OPD		Total		ICU vs. IPD vs. OPD	
	n	%	n	%	n	%	n	%	Chi-squared test	p-value
Candida albicans	154	9.38	198	12.07	98	5.97	450	27.42	4.79	0.091
Candida auris	56	3.41	14	0.85	4	0.24	74	4.51	52.25	<0.001
Candida glabrata	43	2.62	43	2.62	18	1.10	104	6.34	30.40	<0.001
Candida kefyr	4	0.24	1	0.06	0	0.00	5	0.30	4.28	0.118
Candida krusei	96	5.85	125	7.62	55	3.35	276	16.82	0.62	0.734
Candida lusitaniae	0	0.00	0	0.00	1	0.06	1	0.06	4.44	0.109
Candida orthopsilosis	0	0.00	4	0.24	0	0.00	4	0.24	4.83	0.089
Candida parapsilosis	36	2.19	27	1.65	9	0.55	72	4.39	6.37	0.041
Candida pelliculosa	2	0.12	41	2.50	2	0.12	45	2.74	36.64	<0.001
Candida tropicalis	202	12.31	284	17.31	113	6.89	599	36.50	2.68	0.262
Candida utilis	0	0.00	4	0.24	1	0.06	5	0.30	3.15	0.207
Candida rugosa	2	0.12	3	0.18	1	0.06	6	0.37	0.05	0.974
Total	595	36.26	744	45.34	302	18.40	1641	100.00	-	

The distribution of 1,641 fungal isolates across different clinical samples - urine, blood, respiratory specimens, pus, and body fluids - revealed statistically significant differences in species-specific prevalence (p < 0.001 for most comparisons). *Candida tropicalis* was the most frequently isolated species, with the highest proportion from urine samples (n = 375; 22.85%), followed by respiratory specimens (n = 115; 7.01%), with highly significant inter-sample variation (χ² = 97.61; p < 0.001). *Candida albicans*, comprising 27.42% of isolates, was also predominantly recovered from urine (n = 221; 13.47%) and respiratory samples (n = 164; 9.99%), showing a significant inter-sample difference (χ² = 68.20; p < 0.001). *Candida auris* had a notably high representation in blood (n = 54; 3.29%) compared to other sources, reflecting a strong statistical association (χ² = 105.60; p < 0.001). Similarly, *Candida pelliculosa* was found almost exclusively in blood (n = 43; 2.62%), with highly significant distribution (χ² = 133.10; p < 0.001). Other species, such as *Candida glabrata* and *Candida parapsilosis*, also demonstrated significant inter-sample proportions, especially with respiratory and blood samples, respectively. However, isolates like *Candida lusitaniae*, *Candida orthopsilosis*, and *Candida rugosa* showed no statistically significant variation across sample types (p > 0.05), likely due to their low overall frequency. These data underscore that certain *Candida* species have strong sample-type associations, which can guide clinical suspicion and diagnostic strategies in different infection contexts (Table 3).

**Table 3:** Distribution of fungal isolates according to the type of samples n = number, % = percentages

Fungal isolates	Urine sample		Blood sample		Respiratory sample		Pus sample		Body fluid		Total		Inter-sample proportion significance	
	n	%	n	%	n	%	n	%	n	%	n	%	Chi-squared test	p-value
Candida albicans	221	13.47	52	3.17	164	9.99	11	0.67	2	0.12	450	27.42	68.20	<0.001
Candida auris	15	0.91	54	3.29	5	0.30	0	0.00	0	0.00	74	4.51	105.60	<0.001
Candida glabrata	25	1.52	27	1.65	51	3.11	0	0.00	1	0.06	104	6.34	33.79	<0.001
Candida kefyr	1	0.06	4	0.24	0	0.00	0	0.00	0	0.00	5	0.30	9.02	0.061
Candida krusei	111	6.76	65	3.96	97	5.91	2	0.12	1	0.06	276	16.82	13.29	0.010
Candida lusitaniae	1	0.06	0	0.00	0	0.00	0	0.00	0	0.00	1	0.06	1.13	0.890
Candida orthopsilosis	1	0.06	3	0.18	0	0.00	0	0.00	0	0.00	4	0.24	6.09	0.193
Candida parapsilosis	15	0.91	42	2.56	13	0.79	1	0.06	1	0.06	72	4.39	51.95	<0.001
Candida pelliculosa	2	0.12	43	2.62	0	0.00	0	0.00	0	0.00	45	2.74	133.10	<0.001
Candida tropicalis	375	22.85	94	5.73	115	7.01	6	0.37	9	0.55	599	36.50	97.61	<0.001
Candida utilis	1	0.06	1	0.06	0	0.00	0	0.00	3	0.18	5	0.30	170.50	<0.001
Candida rugosa	4	0.24	2	0.12	0	0.00	0	0.00	0	0.00	6	0.37	2.50	0.643
Total	772	47.04	387	23.58	445	27.12	20	1.22	17	1.04	1641	100.00	-	

Table 4 shows the antifungal susceptibility of different *Candida* species. We found antifungal susceptibility patterns of only six *Candida* species: *Candida albicans*, *Candida rugosa*, *Candida pelliculosa*, *Candida parapsilosis*, *Candida krusei*, and *Candida tropicalis*. Fluconazole was found to be the most resistant antifungal across all *Candida* species. *Candida parapsilosis* was found to be 100% susceptible to four antifungals: caspofungin, micafungin, amphotericin B, and flucytosine.

**Table 4:** Shows the antifungal susceptibility of different Candida species

Fungal isolates	Fluconazole n (%)	Voriconazole n (%)	Caspofungin n (%)	Micafungin n (%)	Amphotericin B n (%)	Flucytosine n (%)
Candida albicans	15 (57.7)	20 (76.9)	18 (69.2)	18 (69.2)	17 (65.4)	18 (69.2)
Candida rugosa	1 (25)	4 (100)	1 (25)	1 (25)	2 (50)	3 (75)
Candida krusei	0 (0)	9 (90)	8 (80)	8 (80)	7 (70)	0 (0)
Candida parapsilosis	3 (50)	5 (83.3)	6 (100)	6 (100)	6 (100)	6 (100)
Candida pelliculosa	0 (0)	5 (100)	3 (60)	3 (60)	5 (100)	5 (100)
Candida tropicalis	6 (75)	7 (87.5)	7 (87.5)	7 (87.5)	7 (87.5)	6 (75)

## Discussion

The incidence and antifungal susceptibility profiles of *Candida* infections in hospitalized patients at a tertiary care hospital in North India were studied retrospectively in the current research. Accurate diagnosis and information regarding the distribution of various isolates in each institution are essential for the best possible treatment of candidemia. Hospital infection control and curtailment of its infections in the ICU are necessary, as it is a nosocomial illness with a high mortality rate. The prevalence of nosocomial invasive fungal infections is on the rise. The prevalence of *Candida* infections in our sample was higher in females (n = 945, 57.6%) than in males (n = 696, 42.4%), yielding a ratio of 1.36:1, which is in line with previous research [12]. The existence of a female hormone receptor and improper personal hygiene practices are the causes of the high frequency and aggressiveness of *Candida* in females [13]. However, several studies show that candidiasis is more common in men [14].

In our research, the most commonly affected age group was 55-64 years (n = 434, 26.4%), followed by 65-74 years (n = 318, 19.4%), with a mean age of 55.4 years, which was consistent with other studies [15]. With a ratio of 4.43:1, the highest isolation of *Candida* spp. was seen in admitted patients (wards and ICUs) as opposed to OPD. Numerous factors, including the use of antibiotics, indwelling devices, and immunocompromised patients, may be contributing to this. Urine had the most isolates (n = 772, 47.1%) in the current investigation, followed by respiratory specimens (n = 445, 27.1%). This result is consistent with research by Patel et al. [16].

In our investigation, we found a higher percentage of NAC compared to *Candida albicans*. This finding aligns with other recent research that noted an increase in the percentage of NAC [17]. Epidemiology distribution, diagnostic facilities, and patient types significantly impact the trends of *Candida albicans *and NAC [18]. Increased use of antifungals with improper prescription, short antifungal therapy, and prolonged azole use may explain the shift in frequency from albicans to NAC. This is an alarming concern because NAC has not only surpassed albicans in uncontrolled spread, but this careless use has also developed resistance to azole, polyene, and echinocandin medications [19]. Our findings showed that *Candida tropicalis*, not *Candida albicans*, was the species most frequently isolated from blood. This finding is intriguing since *Candida tropicalis* has become a more common cause of fungemia and can colonize skin. Medical devices and intravascular catheters are the main cause of invasive fungal infections [20].

In a study conducted in Manipal, Mohandas et al. found that 25% of symptomatic urinary tract infection (UTI) patients had *Candida albicans*, and 50% had *Candida krusei* [21]. *Candida tropicalis* was the most prevalent isolate across all samples. The most common species found in respiratory tract samples was *Candida albicans*. Additionally, a 2017 study conducted in Nepal found that 90% of the *Candida* species were detected in urine and sputum, indicating a greater incidence of *Candida* species associated with respiratory tract infection (RTI) and UTI [22].

Compared to urine and other clinical samples, *Candida auris* was most commonly isolated from blood. Longer intensive care unit stays and medical procedures are two identified independent risk factors for acquiring fungal candidemia. Any hospital that encounters *Candida auris*, if not controlled, might cause an epidemic outbreak. It contaminates health care facilities and colonizes due to the formation of biofilm, which helps in spreading among patients in the same wards or intensive care units [23]. Thus, cohorting is essential in wards and ICU settings for the prevention of the spread of this infection. This agent's propensity to persist on inanimate items for a variable period raises the likelihood that it may be transferred to vulnerable patients, ideally by healthcare workers. Prompt action and the use of infection control techniques aid in preventing this fungus from spreading further [24].

The introduction of numerous novel antifungal medications during the recent era has made their efficacy in treating fungal infections imperative. The speciation of *Candida* is necessary to determine which antifungal is best; for example, azoles work well against *Candida albicans* and *Candida tropicalis* but not against *Candida krusei* and *Candida glabrata*. Antifungal sensitivity testing is therefore crucial for controlling isolated strains of *Candida*, especially as NAC species are less vulnerable to amphotericin B and fluconazole [25,26]. Fluconazole resistance is inherent in *Candida* *krusei*, as confirmed in this study [27]. *Candida tropicalis* and *Candida albicans*, on the other hand, showed resistance rates to amphotericin B of (n = 1, 12.5%) and (n = 9, 34.6%), respectively. These results are supported by research by Nazir et al. [28], and Sandhu et al. [29] reported similar resistance levels. Notably, in this investigation, *Candida parapsilosis* exhibited the highest susceptibility to all antifungal groups. Additionally, voriconazole proved to be more efficient than fluconazole; nevertheless, because of their similar chemical structures, voriconazole resistance is increasing as a result of cross-resistance. In 11.3% of isolates, Oberoi et al. observed cross-resistance and decreased sensitivity to both fluconazole and voriconazole [30].

Limitations

The present study's limitations include the lack of additional tests for species confirmation. We did not correlate clinical outcomes due to a lack of data. Furthermore, the data came from a single institution and cannot be extended to other hospitals.

## Conclusions

In our tertiary care facility, *Candida tropicalis* is the most commonly isolated pathogen. NAC and *Candida albicans* both exhibit decreased susceptibility to azole antifungals, particularly fluconazole, although NAC's patterns of decreased susceptibility to these medications are more noticeable. The changed susceptibility patterns of NAC species to a number of antifungal medications frequently used in clinical practice are a major source of worry. As a result, it is critical to keep a careful eye on the prevalence of various species and how susceptible they are to antifungals.
